# Charge Profile Analysis Reveals That Activation of Pro-apoptotic Regulators Bax and Bak Relies on Charge Transfer Mediated Allosteric Regulation

**DOI:** 10.1371/journal.pcbi.1002565

**Published:** 2012-06-14

**Authors:** Crina-Maria Ionescu, Radka Svobodová Vařeková, Jochen H. M. Prehn, Heinrich J. Huber, Jaroslav Koča

**Affiliations:** 1 CEITEC - Central European Institute of Technology, Masaryk University, Brno, Czech Republic; 2 National Centre for Biomolecular Research, Masaryk University, Brno, Czech Republic; 3 Centre for Systems Medicine, Royal College of Surgeons in Ireland, Dublin, Ireland; 4 Department of Physiology and Medical Physics, Royal College of Surgeons in Ireland, Dublin, Ireland; University of Houston, United States of America

## Abstract

The pro-apoptotic proteins Bax and Bak are essential for executing programmed cell death (apoptosis), yet the mechanism of their activation is not properly understood at the structural level. For the first time in cell death research, we calculated intra-protein charge transfer in order to study the structural alterations and their functional consequences during Bax activation. Using an electronegativity equalization model, we investigated the changes in the Bax charge profile upon activation by a functional peptide of its natural activator protein, Bim. We found that charge reorganizations upon activator binding mediate the exposure of the functional sites of Bax, rendering Bax active. The affinity of the Bax C-domain for its binding groove is decreased due to the Arg94-mediated abrogation of the Ser184-Asp98 interaction. We further identified a network of charge reorganizations that confirms previous speculations of allosteric sensing, whereby the activation information is conveyed from the activation site, through the hydrophobic core of Bax, to the well-distanced functional sites of Bax. The network was mediated by a hub of three residues on helix 5 of the hydrophobic core of Bax. Sequence and structural alignment revealed that this hub was conserved in the Bak amino acid sequence, and in the 3D structure of folded Bak. Our results suggest that allostery mediated by charge transfer is responsible for the activation of both Bax and Bak, and that this might be a prototypical mechanism for a fast activation of proteins during signal transduction. Our method can be applied to any protein or protein complex in order to map the progress of allosteric changes through the proteins' structure.

## Author Summary

Apoptosis is a physiological form of cell death that is fundamental for development, growth and homeostasis in multi-cellular organisms. Deviations in the apoptosis machinery are known to be involved in cancer, neurodegenerative disorders, and autoimmune diseases. The proteins Bax and Bak are essential for executing apoptosis, yet the mechanism of their activation is not properly understood at the structural level. To understand this mechanism, we investigated how the electronic density is reorganized (i.e., how charge is transferred) inside the Bax molecule when Bax binds a functional peptide of its natural activator protein. We identified the specific interactions responsible for the exposure of the functional sites of Bax, rendering Bax active. Furthermore, we found a network of charge transfer that conveys activation information from the Bax activation site, through the hydrophobic core of Bax, to the well-distanced functional sites of Bax. This network consists of three residues inside the hydrophobic core of Bax, which are present also in the hydrophobic core of Bak, suggesting that these residues are functionally important and thus potential drug targets. We provide a straightforward and accessible methodology to identify the key residues involved in the fast activation of proteins during signal transduction.

## Introduction

Mitochondrial outer membrane permeabilization (MOMP) is a hallmark of programmed cell death (apoptosis). Following MOMP, apoptotic proteins from the mitochondrial inter-membrane space are released, causing the activation of cell death proteases which cleave the cell's cytoskeleton and genetic material. MOMP is executed by the Bcl-2 family proteins Bak and Bax that, upon activation during apoptosis, oligomerize and form pores in the mitochondrial membrane [1]–[4].

Bak and Bax oligomerisation is controlled by the interplay of further Bcl-2 proteins [5]–[8]. While pro-survival Bcl-2 proteins bind to and deactivate Bak and Bax [9], other apoptotic Bcl-2 proteins de-repress this inhibition, leaving Bak and Bax free to oligomerize [10]. Nevertheless, a separate step, whereby a subclass of apoptotic Bcl-2 proteins such as Bim and Bid directly activate Bak and Bax, was proposed to be required for oligomerization [11]–[13].

**Figure 1:**
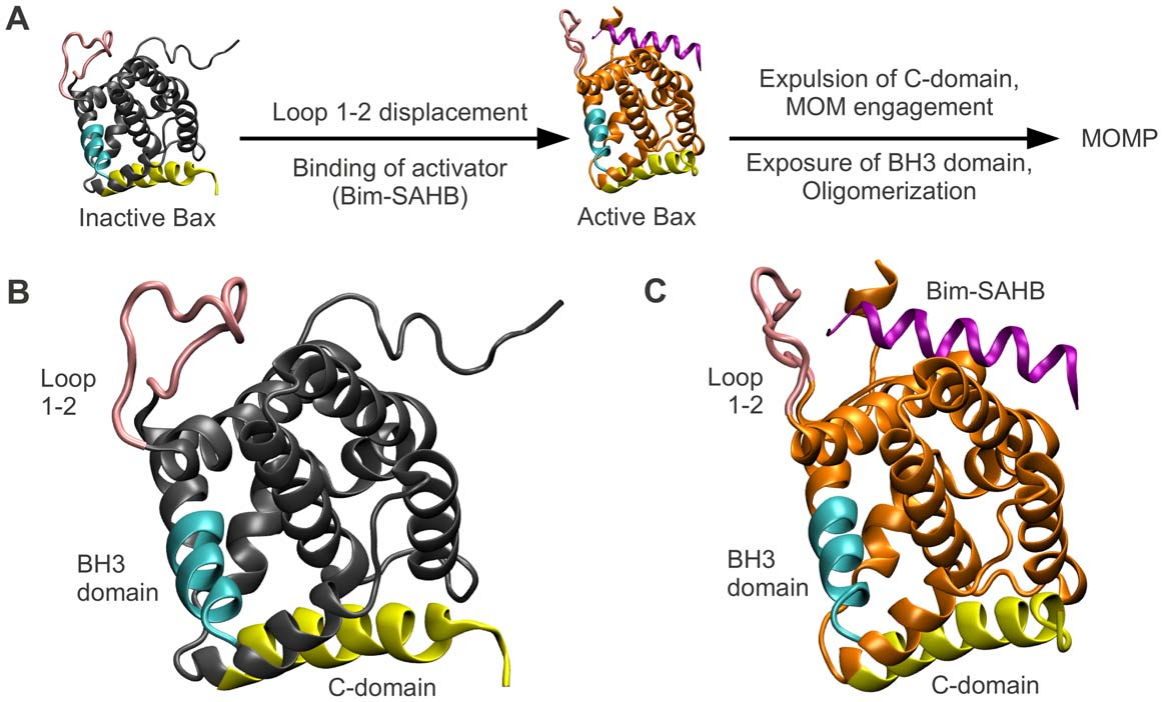
Bax undergoes several conformational changes enabling it to form pores in the mitochondrial outer membrane. (A) Bax activation leads to mitochondrial outer membrane permeabilization (MOMP). Inactive Bax is a cytosolic monomer. Activator-induced opening of loop 1–2 allows the activator to bind. Subsequently, the C-domain of the now active Bax vacates the BH groove and inserts into the mitochondrial membrane. Additionally, the Bax BH3 domain gets exposed. Bax oligomerization ensues via the BH groove and the BH3 domain, eventually leading to the formation of pores which permeabilize the membrane. (B) Location of the BH3 domain (cyan), the C-domain (yellow), loop 1–2 (pink) in inactive Bax (the rest of the protein in gray). (C) Location of above domains (same color coding) in active Bax (the rest of the protein in orange). The activator peptide Bim-SAHB is shown in purple.

The activation steps required for Bax oligomerization were extensively investigated [14]–[18]. These steps were found to comprise Bax translocation from the cytosol to the mitochondrial membrane, and changes of Bax conformation. Conformational changes of Bax include exposure of its C-domain, insertion of this C-domain into the membrane, and exposure of the Bax BH3 domain, one of four homology domains of Bcl-2 proteins (Figure 1).

In inactive Bax, the C-domain is tightly bound inside a hydrophobic pocket which we henceforth denote as the ‘BH groove’. This tight binding was suggested to increase the solubility of Bax and to keep Bax in the cytosol in the absence of stress [16]. Gavathiotis et al. [18] synthesized a helix mimicking the BH3 domain of the activator Bim (Bim-stabilized α-helix of Bcl-2 domains, Bim-SAHB). They subsequently performed NMR spectroscopy to study the interaction of Bax with the Bim-SAHB activator. They found that, in the absence of Bim-SAHB, the Bax activation site was blocked by a largely unstructured loop (loop 1–2), which opens upon incubation with Bim-SAHB. Using Bax mutants with reduced loop 1–2 mobility, Gavathiotis et al. later demonstrated that the opening of this loop was a prerequisite for Bax activation [19]. Interestingly, the suggested Bax activation site and the Bax C-domain are separated by over 25 Å. Since the binding of Bim-SAHB to Bax is weak and transient, and neither significant disturbances in the helical packing, nor covalent modifications have been observed in Bax upon activation, the mechanism of how C-domain exposure occurs following this activation remains elusive [20], [21].

Charge transfer was found to be significant in many biomolecular interactions [22]–[24], and functionally linked to protein structural dynamics [25]. In this paper, we therefore investigate the role of charge transfer during Bax activation by employing an electronegativity equalization model for the calculation of atomic charges. Following our investigation, we propose that a charge transfer network is intimately connected to the way that the activation information travels across Bax, and that a similar network is plausible in Bak.

## Results

### Calibration of an EEM Model for Calculating Partial Atomic Charges in Proteins

The Electronegativity Equalization Method (EEM) [26] is a fast technique for estimating partial atomic charges, and has been successfully applied to zeolites, small organic molecules and polypeptides [27]–[31]. To use EEM for studying charge transfer during Bax activation, EEM model parameters need to be calibrated to charges of reference molecules.

**Figure 2:**
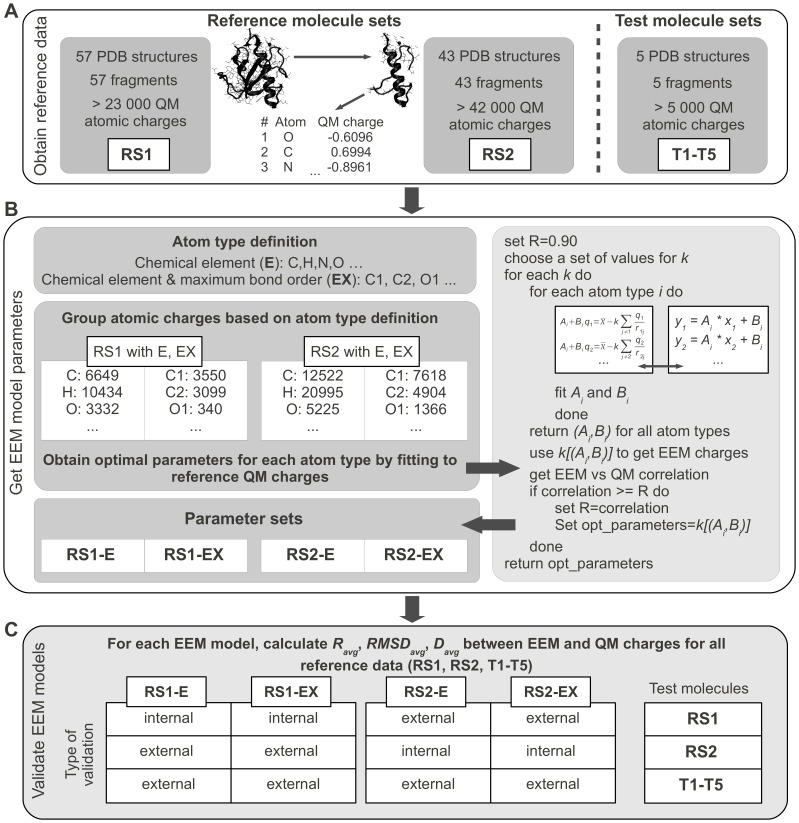
Flowchart of calibration of an EEM model for calculating partial atomic charges in proteins. (A) The reference data used in this study consisted of QM atomic charges for protein fragments in two reference sets (RS1, RS2) and one test set (T1–T5). (B) Two atom type definitions were used. The atomic electronegativity equations were grouped together based on the atom type. The EEM model parameters for each atom type were then obtained by least squares fitting to reference QM charges. (C) Each EEM model was subjected to internal and external validation by comparing the EEM charges with reference QM charges for all available data sets (RS1, RS2, T1–T5).

For this purpose we followed our previously published EEM model calibration procedure [32], with a few modifications that address the complex nature of proteins. The reference data consisted of atomic charges for molecules of two disjoint reference sets RS1 and RS2, and these charges were calculated using the quantum mechanics (QM) scheme detailed in the Methods section. Since calculation of QM charges for large molecules such as proteins would require too high computational costs, previous EEM models available in literature were calibrated to reference charges from small molecules [32]–[38]. Moreover, to make EEM as generally applicable as possible, these calibrations used mostly inorganic or drug-like compounds, which do not reflect the complex nature of proteins as long, non-neutral molecular chains with complex 3D assembly. Therefore, to retain properties that are characteristic for proteins and allow a fast calculation of reference QM charges at the same time, large fragments of experimentally determined protein structures were used as reference sets in the present study (see the Methods section for details). We next determined values for the EEM model parameters by fitting them to the reference QM charges using a least squares algorithm. Prior to fitting, we classified atoms according to two schemes. One scheme was based on chemical elements only (denoted ‘E’), and the other on chemical elements and maximum bond order for each atom (denoted ‘EX’, so that, for example, ‘O1’ indicates simple bonded, and ‘O2’ double bonded oxygen). Fitting the model parameters for each of the two atom classification schemes and each of the two reference sets of atomic charges, we obtained four parameter sets, denoted RS1-E, RS1-EX, RS2-E, RS2-EX. Finally, we validated our EEM model by assessing the accuracy of the model in reproducing the original QM charges from reference sets RS1 and RS2, and from five additional test molecules T1-T5. Results were evaluated by the average correlation coefficient *R_avg_* (squared Pearson's correlation coefficient), the root mean square deviation *RMSD_avg_*, and the average absolute difference *D_avg_*. An overview of the EEM model calibration procedure is given in Figure 2, and the complete details can be found in the Methods section.

**Figure 3:**
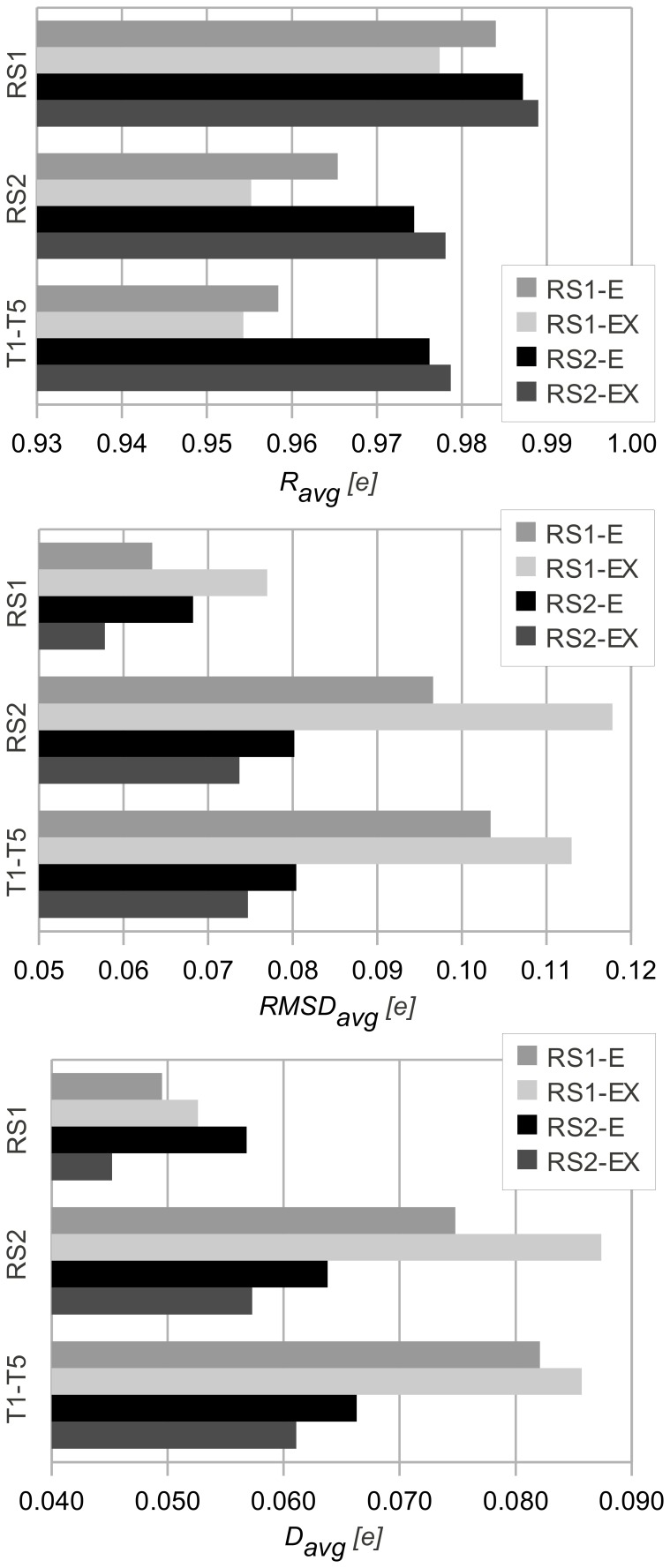
Validation of EEM models by comparing EEM atomic charges against QM atomic charges. Statistical descriptors comprising the average correlation coefficient (*R_avg_*), the average root mean square deviation (*RMSD_avg_*) and the average absolute difference (*D_avg_*) are given. These descriptors quantify the agreement between EEM model charges and QM charges for molecules belonging to the reference sets RS1 and RS2, and for five further test molecules T1–T5. All quantities are given in elementary charges (1 *e*∼1.602×10^−19^ coulombs). The names of the parameter sets encode the reference set and atom classification scheme based on which they were developed (RS1-E, RS1-EX, RS2-E, RS2-EX). Good agreement between QM and EEM charges was found for all data sets, as *R_avg_* is close to 1, and *RMSD_avg_* and *D_avg_* are minimal. Calibrations that used the coarse atom type classification ‘E’ gave a similarly good agreement as those where the more detailed classification scheme ‘EX’ was used.

Overall, the results in Figure 3 suggested that the finer grained atom classification scheme ‘EX’ only modestly improved the accuracy compared to the scheme ‘E’ based on chemical elements alone. The good agreement between QM and EEM charges for all data sets suggested that both atom classification schemes can provide satisfactory calibration results. Moreover, our model was able to compute EEM atomic charges in less than 1 second for any of the reference or test molecules using our previously published implementation [39].

### Bax Is Activated by Arg94-mediated Abrogation of the Ser184-Asp98 Interaction, Decreasing the Affinity of the Bax C-domain for Its Binding Groove

Having developed an EEM based method for rapid calculation of atomic partial charges, we investigated whether atomic charge distribution prior and subsequent to Bax activation would reveal any clues about the mechanisms of the activation. To this end, we obtained the 3D structure of inactive Bax (Figure 1B), and of active Bax in complex with the activator peptide Bim-SAHB (Figure 1C) from the Protein Data Bank (PDB IDs 1F16 [16] and 2K7W [18] respectively). We then computed EEM atomic charges using parameter set RS2-E (Figure 2B) for both structures, and assessed the absolute charge transfer per residue (total difference in charge per amino acid residue, *ΔQ_res_*), and the intra-residue charge density reorganization (root mean square deviation in charge per residue, *RMSD_res_*). The mathematical derivation of these descriptors can be found in the Methods section, and their values for all Bax residues are available in Table S1.

**Figure 4:**
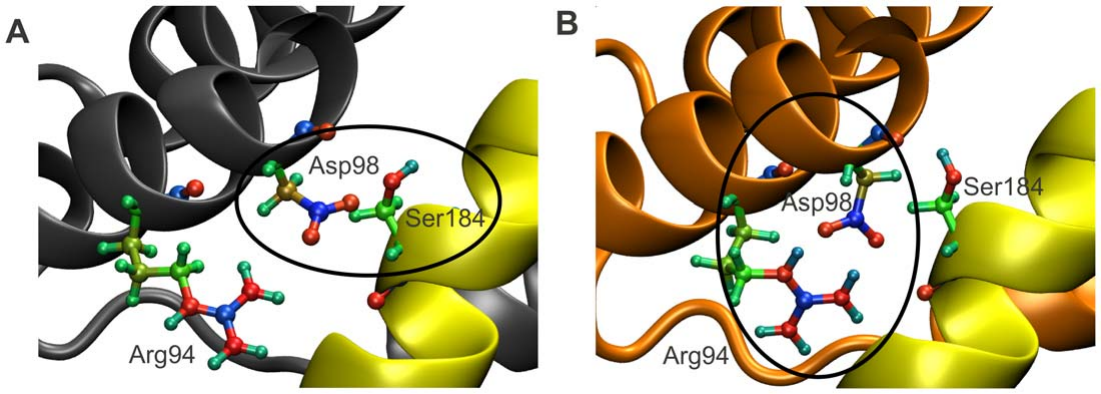
In active Bax, Arg94 recruits Asp98, destabilizing the C-domain inside the BH groove. Upon activation, Arg94 becomes more positive, leading to the recruitment of Asp98, abrogation of the Asp98-Ser184 interaction, and ultimately destabilization of the C-domain inside the BH groove [16], [40]. The color coding from Figure 1 is maintained. Additionally, the atoms in residues Arg94, Asp98 and Ser184 are displayed explicitly. Colors are coded according to their EEM charges, where the color scale ranges from red, through green, to blue, as values of atomic charges go from negative to positive. The EEM charges were computed using parameter set RS2-E (see Figures 2 and 3). (A) In inactive Bax, Asp98 is engaged in an interaction with Ser184, which keeps the C-domain in its binding pocket. (B) In active Bax, the now more positively charged Arg94 (see also Table S1) has sequestered Asp98, which no longer contributes to the stabilization of the Bax C-domain in its BH groove.

Experimental evidence suggests that, in inactive Bax, the C-terminal helix is bound tightly to its hydrophobic pocket (‘BH-groove’). During activation, this binding gets destabilized, causing the C-domain to subsequently vacate the BH-groove and insert into the mitochondrial outer membrane. Early mutagenesis studies revealed a critical interaction between residues Ser184 and Asp98 at the C-domain-BH-groove interface, whose abrogation is sufficient to immediately activate Bax [16], [40]. We therefore focused on the changes in charge density distribution in the vicinity of this interaction. While our calculations did not show any change in the charge profile of Ser184, they indicated that any interaction that might have taken place between Asp98 and Ser184 in the inactive structure has been replaced by an Asp98-Arg94 salt bridge in the active structure (Figure 4). Upon activation, Arg94 becomes more positive (see also Table S1), which is suggested to lead to the recruitment of Asp98, the abrogation of the Asp98-Ser184 interaction, and ultimately the destabilization of the C-domain. This demonstrates that the binding of Bim-SAHB to Bax can activate Bax by destabilizing the interaction between the Bax C-domain and its binding groove.

### A Network of Charge Transfer Extends from the Bax Activation Site, through Its Hydrophobic Core, to the C-domain Binding Groove

It remains puzzling how the BH groove is influenced by the binding of Bim-SAHB to Bax, given that this interaction occurs on the opposite side of the Bax molecule, at a distance of 25 Å from the BH groove.

Interestingly, the residues which showed a transfer of charge one standard deviation higher than average (Table S1) provided a clue as to how the activation information proceeds through the protein. Foremost, significant changes in the net residue charges were found at the Bax activation site, the BH3-domain (required for oligomerization) and the C-domain (required for membrane insertion). Since these are all functional sites of Bax, these changes were not unexpected. For example, George et al [41] found that a triple alanine mutant at residues 63–65 (on the BH3 domain of Bax) ablated Bax oligomerisation and apoptotic activity, which correlates perfectly with the high charge transfer we found on residues 64 and 65 upon Bax activation (Table S1).

**Figure 5:**
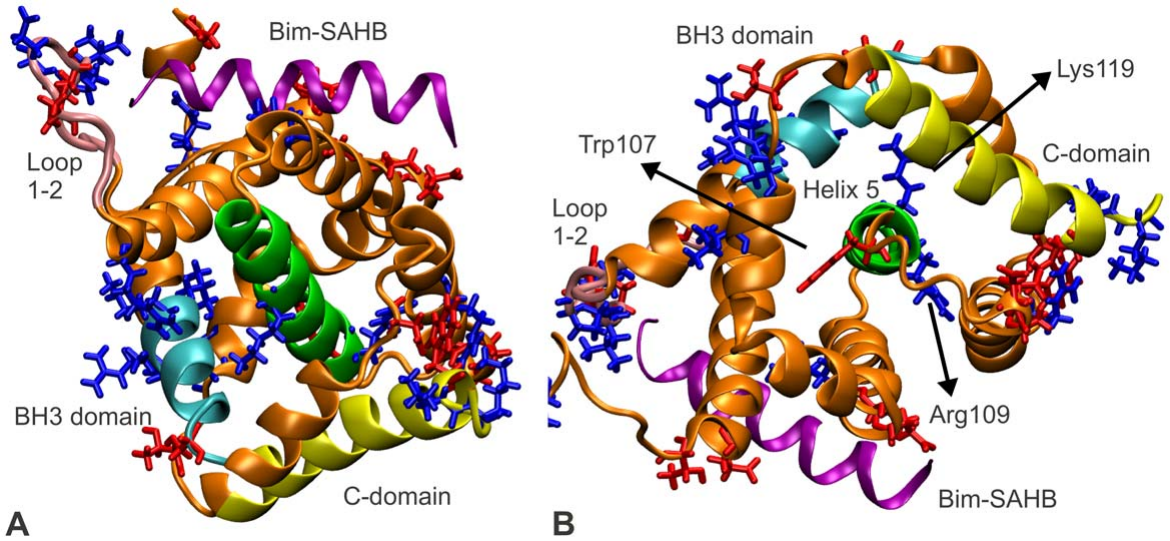
Proposed charge transfer network in Bax, indicated by net changes in residue charges. The information of the Bim-SAHB induced activation of Bax is transmitted from the Bax activation site via a charge transfer network through the core of the Bax protein, up to the Bax C- and BH3-domains. Inside the hydrophobic core of Bax, the central helix, helix 5, acts as a hub which collects and distributes charge density, mainly through residues Trp107, Arg109 and Lys119. The color coding from Figure 1 is maintained. Additionally, helix 5 is highlighted in green. The Bax residues which transfer an amount of charge of one standard deviation higher than average (Table S1) are explicitly displayed and color coded according to whether they become more positive (blue) or negative (red) upon activation. (A) The residues which transfer a significant amount of charge were found at the Bax activation site, on the loop 1–2, inside the BH groove holding the Bax C-domain, and at one end of the C-domain (see also Figure S1). Additionally, several such residues were found on helix 5, the central helix in Bax, and on the Bax BH3 domain, suggesting that the interaction at the Bax activation site is transmitted via a network of charges from the activation site, through the protein core, to the C- and BH3-domains. (B) Top view of helix 5 is given. The organization of residues Trp107, Arg109 and Lys119 inside the hydrophobic core of Bax suggests that helix 5 acts as a charge transfer hub, which integrates and distributes charge density.

However, in addition to the expected changes, our method surprisingly identified significant charge transfer also on the central helix, inside the hydrophobic core of Bax (residues Trp107, Arg109 and Lys119 on helix 5). The presence of significant charges and charge transfer in a predominantly hydrophobic environment suggests that helix 5 acts as a hub which collects and distributes charge density (Figure 5). We further calculated the intra-residue redistributions of charge density upon activator (Bim-SAHB) binding. Significant such redistributions were observed at the Bax activation site, BH groove and loop 1–2. Since these are the functional regions of Bax, these calculations provide further support for the notion of a charge transfer network that conveys information across the entire Bax molecule (Figure S1, Table S1).

Hints of such an interaction transfer phenomenon were found by Gavathiotis et al. [19]. They titrated Bax with increasing amounts of Bim-SAHB and observed small, but reproducible dose-responsive changes in NMR resonance behavior for the backbone N atoms of residues on the Bax C-domain, as well as on helix 5 inside the hydrophobic core of Bax. They concluded that the binding of the activator induces reverberations in the core of the Bax protein, which serve to mobilize the C-domain (allosteric sensing). Our charge analysis explains these reverberations by a network of charge transfer through the entire Bax molecule.

### The Residues Essential for the Charge Transfer Network in Bax Are Conserved in Bak

**Figure 6:**
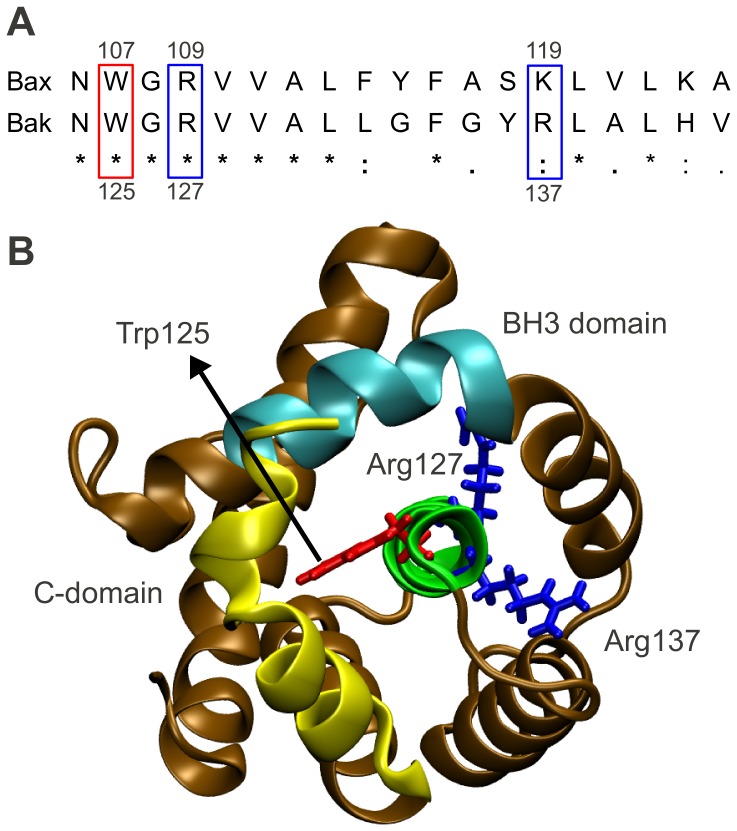
Proposed Charge Transfer Network in Bak. (A) Sequence alignment of central helices of Bak and Bax. An asterisk indicates a single, fully conserved residue. A colon indicates conservation between groups of strongly similar biochemical properties. A period indicates conservation between groups of weakly similar biochemical properties. The residues involved in the charge transfer network in Bax are conserved in Bak as Trp125, Arg127 and Arg137. (B) Bak structure (ochre) is displayed according to Figure 5B, with the same top view of the central helix, and the same color coding for C-domain (yellow), BH3-domain (cyan), central helix (green) and hub residues (red and blue). Residues Trp125, Arg127 and Arg137 are organized in a similar manner to their Bax homologues, suggesting that they may also play an essential role during Bak activation.

Unlike Bax, Bak is present at the outer mitochondrial membrane in absence of apoptotic stimuli. Evidence suggests that the inactive form of Bak gets recruited to the mitochondrial outer membrane and forms complexes with the membrane protein VDAC2. Upon apoptotic stimuli, pro-apoptotic Bcl-2 proteins such as Bid transiently bind to Bak. This binding breaks down the VDAC2/Bak complex and exposes the BH3 domain of Bak, which is essential for Bak oligomerization [42]–[46]. As the activation information may be conveyed by a similar charge transfer network to induce abrogation of VDAC2/Bak binding, we wondered whether a comparable transfer hub may exist also in Bak. Since residues that are essential for functionality are most often conserved in proteins with similar functions, we therefore first performed the sequence alignment of Bax and Bak. While the sequence identity between the two proteins was rather low (ClustalW2 score 19%, see Figure S2), we found that the residues involved in the charge transfer network in Bax were conserved in Bak. These homologous residues were Trp125, Arg127 and Arg137 (Figure 6A). We subsequently compared the 3D structures of Bax and Bak (PDB ID 2IMT [47]), and found that above Bak residues were organized in a very similar manner to their Bax homologues (Figures 5B and 6B). These findings suggest that the mechanism of charge transfer via the hydrophobic core of Bax is also plausible for Bak, and that similar residues may also play an essential role during Bak activation.

## Discussion

Allosteric proteins are characterized by a regulatory site that is distinct and often well distanced from the protein's active site. Regulation of the protein's activity which occurs via this distinct site is termed allosteric regulation. Recent reports indicate that allosteric regulation is particularly important during cell signaling processes, where it has been shown to stabilize receptor proteins, or to be responsible for the rapid, stress induced release of dormant signaling proteins bound to the cytoskeleton [48], [49]. An interesting structure-function analysis of Bax performed by George et al. [41] concluded that monomeric Bax may be held in an inactive conformation by multiple helices in the absence of stress, and that Bax may be activated through perturbation at multiple sites. Nevertheless, later Gavathiotis et al. identified a unique and well defined activation site on Bax [18], and subsequently demonstrated that binding of an activator BH3 peptide induces reverberations in the core of the Bax protein, a phenomenon they named allosteric sensing [19]. The present study found that this allosteric regulation is mediated by a charge transfer network, which conveys the activation information from the Bax activation site to the functional regions of Bax without compromising the structure of the BH groove (essential for pro-apoptotic activity). As charge transfer is significantly faster than domain rearrangements, the charge transfer mediated alosteric regulation in Bax also allows for a swift control of the apoptotic fate [50].

In addition to suggesting that charge transfer mediated allosteric regulation is responsible for Bax activation by pro-apoptotic Bcl-2 proteins, our charge profile analysis also indicated several residues that actively mediate this charge interaction, providing an opportunity for further in-depth mutagenesis studies or even pharmacological intervention.

We first confirmed that the abrogation of the Asp98-Ser184 interaction, which has been reported to be responsible for the mobilization of the C-domain from the BH groove [16], [40], indeed occurs during Bax activation. We propose that Arg94 plays an essential role in this abrogation, as it can sequester Asp98 and prevents the formation of the stabilizing Asp98-Ser184 interaction in active Bax. Indeed, previous mutational studies [41] showed that a triple alanine Bax mutant at residues 92 to 94 is biologically inactive, supporting our finding that residue Arg94 plays a role in Bax activation.

Furthermore, we found that helix 5 acts as a central hub for the charge transfer network in Bax. We identified three residues, Trp107, Arg109 and Lys119, that may act as the main mediators of this charge transfer. Helix 5 has been found to react to Bim-SAHB binding in NMR experiments [19]. It was then found that the Bim-SAHB-induced opening of the Bax loop 1–2 is essential for Bax activity, and that this opening induces reverberations in the protein's hydrophobic core. A deeper look at the NMR data from their supplement (Figure S1D from [19]) reveals that activator binding induces pronounced chemical shifts in the Bax backbone N atoms in the area of residue Trp107 even when the mobility of loop 1–2 is restricted by chemical tethering. In Figure S1B from the same publication [19], we observe that opening this loop similarly affects the backbone N atoms in the vicinity of Lys119. While the authors [19] did not explicitly focus on these residues, our charge calculations make it reasonable to assume that they are indeed important for allosteric Bax activation. Moreover, another study [41] found that triple alanine Bax mutants at residues 109–111 or 118–120 showed decreased biological activity in the presence of the activator tBid. Therefore, influencing the activity of Trp107, Arg109 or Lys119 may readily influence the biological activity of Bax. Because of their positioning, residues Trp107 and Arg109 are easily accessible and therefore excellent drug targets.

The results of our investigation agree well with the mutational study of George et al [41], in that helix 5 is a central mediator of Bax activity. Both studies further agree that Arg94 is essential for Bax oligomerisation, and that residues Arg109 or Lys119 may influence the biological activity of Bax. In addition, George et al. suggested that the block of four central residues (113–116) is mandatory for Bax activity. Comparatively, the amount of charge transferred by these 4 residues upon Bax activation was only slightly above average (Table S1). Nevertheless, we note that this block of residues resides at the centre of the Bax molecule and is very bulky, and therefore these residues are very likely essential for maintaining the helical fold of Bax. Therefore, the observation of George et al. that a quadruple mutation in the centre of the Bax molecule impairs biological activity can be easily explained by change of stability of the helical fold, rather than by a disruption of the Bax activation mechanism.

Finally, by performing sequence and structural alignment of Bax and Bak, we identified that Bak residues Trp125, Arg127, and Arg137 potentially constitute a similar hub of charge transfer inside the Bak protein. The membrane protein VDAC2 was reported to recruit Bak to the mitochondrial membrane in the absence of apoptotic stimuli. Upon apoptotic stimuli, this VDAC2/Bak binding can be abrogated by Bcl-2 proteins that transiently bind to Bak [42]–[46]. Cheng et al. [42] showed that Bak mutations at residues Leu78 (within Bak's BH3 domain), Trp125, Gly126 and Arg127 (on Bak's central helix) impair complex formation with VDAC2, and thus concluded that VDAC2 binding to Bak must occur in the proximity of the above mentioned residues. Having identified those residues that may constitute a transfer charge hub during Bak activation, we propose a charge transfer mediated allosteric activation mechanism of Bak that is similar to that of Bax. We propose that transient activator binding in the vicinity of Arg137 is transmitted through allosteric sensing to the VDAC2 binding site, which is in the neighborhood of Trp125 and Arg127. Subsequently, Bak disengages VDAC2, exposes its BH3 domain and oligomerizes. Since the relevance of residue Arg137 has not been assessed to date, its investigation may further our understanding of the details of Bak activation and its de-repression of VDAC2.

Understanding the structural changes of Bax and Bak during apoptosis provides important insights into the mechanisms of Bax and Bak activation, which steps and key players are involved, how aberrant protein folding or mutations may influence the protein's function, and how their activation may be influenced by inhibitors or synthetic drugs. While X-ray crystallography and NMR spectroscopy provide excellent experimental techniques to obtain 3D-structures, further theoretical data analysis tools are needed to obtain better mechanistic and functional insights into the structural aspects of protein activation. We report here the first successful application of the Electronegativity Equalization Method to study protein activation during programmed cell death, which enabled us to detect and track the allosteric effects responsible for Bax activation by BH3-only proteins. We have thus shown how knowledge of atomic charges can yield insight into biological phenomena even without further simulations or intricate computations. Moreover, the methodology we developed is directly applicable to other molecular systems, and thus of interest in biomedical and pharmacological research.

## Methods

### EEM Formalism

The Electronegativity Equalization Method (EEM) [26] was employed here to estimate the charge transfer upon Bax activation using structural data obtained from the Protein Data Bank (PDB). EEM enables the determination of connectivity- and geometry- dependent atomic charges. Various formalisms are available in literature [32]–[38], [51]–[53]. In the present implementation, we focused on the original work by Mortier et al. [26] with a minor modification as suggested by Yang and Wang [54] and described below. Besides enabling a fast and versatile calibration, this formalism estimates atomic charges via a set of coupled linear equations which can be efficiently solved by a Gaussian elimination procedure (see below).

EEM is based on the Electronegativity Equalization Principle [55], which was proven within the Density Functional Theory [56] and which states that electronegativity 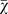 is equalized throughout a molecule (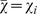). The electronegativity 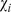 of each atom *i* in this molecule can be approximated as a linear function of several terms:
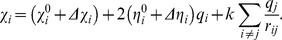



The first term is the effective electronegativity (i.e., the electronegativity 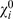 of the neutral atom, corrected for the presence of the molecular environment 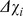). The second term is the charge of the atom *q_i_* multiplied by its effective hardness (i.e., the hardness 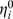 of the neutral atom corrected for the presence of the molecular environment 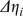). Hardness was defined by Parr and Pearson [57] as the second derivative of the energy with respect to the charge, and can be thought of as the resistance to change in charge. The last term 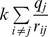 accounts for the electrostatic interaction with every other charged atom *j* in the molecule. *k* is an adjusting factor first introduced by Yang and Wang [54].

Setting 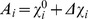 and 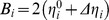, the molecular electronegativity can be written as:
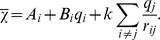



Considering the total molecular charge *Q* to be the sum of all partial atomic charges *q_i_* (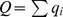), a system of equations results, from which the partial atomic charges *q_i_* and the molecular electronegativity 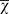 can be calculated:
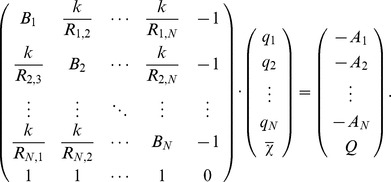



### Calibration of the EEM Model

The corrections for electronegativity 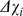 and hardness 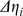 cannot be measured [26]. Therefore, the effective electronegativity and hardness contributions given by 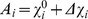 and 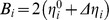 respectively were calibrated in this study. The additional parameter *k* was also determined, as it allows for a computationally cheap and straightforward sampling of the *(A,B)* parameter space, as previously demonstrated by Svobodová Vařeková et al. [32].

The EEM equation of the molecular electronegativity can be rearranged as a linear equation in *A* and *B* for each atom in the system:
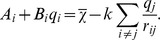



The above linear equations can be grouped together according to the type of atom they refer to, as each parameter will be valid only for a particular type of atom. The classification of atoms into types can be done according to various criteria. As schemes in literature use different levels of granularity [e.g.], [ 32], [33,35], two schemes of atom classification were tested in the present study (see also the Results section).

For each atom type, the parameters *A* and *B* can be determined by least squares minimization, provided that the values of all the other variables in the equation are known. Here the interatomic distances were calculated from the 3D atomic coordinates. The reference atomic charges were calculated by the QM scheme described below. For each molecule, the value of the global electronegativity was approximated as the harmonic average of the electronegativities of its constituent atoms [58]:
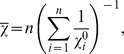
where n is the number of atoms in the molecule, and the values of 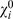 correspond to Pauling electronegativities [59], [60]. The extra parameter *k* present in this particular formalism was sampled on several intervals. For each discrete value of *k*, the least squares minimization was performed in order to obtain the *(A,B)_x_* parameters, where *x* goes over all atom types considered. Upon internal validation, the result was the set of parameters *[k,(A,B)_x_]* which gives the best *R_avg_* (see below) between the reference QM values and the predicted EEM values for atomic charges. A scheme of the calibration step is given in Figure 2B, while a detailed description of this procedure can be found in the work of Svobodová Vařeková et al. [32].

### Reference Data Used for EEM Model Calibration

Obtaining appropriate reference data is essential for the accuracy and applicability of a predictive model. The reference data used in this study consisted of atomic charges for molecules of two disjoint reference sets RS1 and RS2, and these charges were calculated using quantum mechanics (QM) (see below). These reference molecules were fragments extracted from calcium containing proteins which were obtained from PDB and whose structures had been determined by X-ray crystallography or solution state NMR experiments. Each of the fragments consisted of amino acid chains, calcium ions and water molecules, and was obtained from the 3D structure of its parent protein using the program Triton [61]. The fragments were curated manually to ensure that, while they are sufficiently small for QM calculations, they remained biochemically meaningful. For each fragment, reference QM atomic charges were obtained from a Mulliken population analysis performed at the HF/6-31G* theory level using the program Gaussian 03 [62].

An overview of the composition of all fragments used for EEM model calibration is given in Table 1, while the 3D structures of these fragments are available online in PDB format at www.ncbr.muni.cz/~ionescu/Supporting_Data_Sets.zip. Reference sets RS1 and RS2 were used for model calibration, and internal and external validation (see below). Five additional test molecules T1-T5 were used for external validation. A brief summary regarding the reference data is given in Figure 2A.

**Table 1:** Summary of the atomic composition of all the protein fragments used for EEM model calibration.

System	RS1	RS2	T1	T2	T3	T4	T5
**Atoms**	23259	42295	1167	1125	1065	1040	1075
**Fragments**	57	43	1	1	1	1	1
**C**	6649	12522	350	342	305	298	260
**H**	10434	20995	562	505	498	503	558
**N**	2730	3339	117	129	123	104	75
**O**	3332	5225	136	144	136	133	176
**S**	30	149	0	3	2	0	6
**Ca**	84	65	2	2	1	2	0

Reference sets RS1 and RS2 were used for model calibration, and internal and external validation. Five additional test molecules T1–T5 were used for external validation.

### Validation of the EEM Model

The accuracy of the EEM models in reproducing the original QM charges from reference sets RS1 and RS2, and from five additional test molecules T1–T5 was evaluated by internal and external validation. In the internal validation step, the charges predicted by the EEM model with parameter sets RS1-E and RS1-EX (RS2-E and RS2-EX respectively) were compared against QM charges from the associated reference set RS1 (RS2 respectively). In the external validation step, EEM and QM charges were compared for five test molecules T1–T5 which were not contained in the original reference sets RS1 and RS2, but were obtained in a similar manner. Since the reference sets RS1 and RS2 were disjoint, two further external validations were performed. Therefore, EEM charges obtained by using parameter sets RS1-E and RS1-EX (RS2-E and RS2-EX respectively) were compared against QM charges from the non associated reference set RS2 (RS1 respectively). A schematic representation of the EEM model validation step is given in Figure 2C.

The correlation between the sets of QM and EEM charges was assessed by three indicators. The first indicator was the average correlation coefficient (squared Pearson's correlation coefficient), computed for each molecule, and averaged over all molecules in a set:
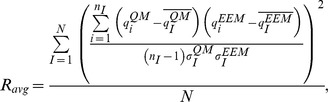
where the index *i* = 1 …N described all atoms in molecule *I*, 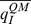 and 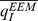 represented average atomic charges in molecule *I*, 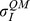 and 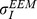 were standard deviations of the atomic charges in molecule *I*, *n_I_* was the number of atoms in molecule *I*, and *N* was the number of molecules in a given set.

The second indicator was the root mean square deviation, computed for each molecule, and averaged over all molecules in a set:
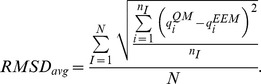



The third indicator was the average absolute difference, computed for each molecule and averaged over all molecules in a set:
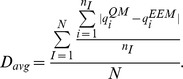



### Evaluating Differences in Charges upon Bax Activation

The EEM charge calculations for both Bax structures were done using the program EEM_SOLVER [39] which implemented the above mentioned EEM formalism and employed the parameter set RS2-E developed in the present study.

Two indicators were employed in order to quantify the changes in the charge profile of Bax upon activation. The first indicator was the total difference in charge per amino acid residue:
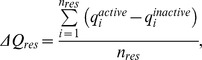
where 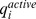 denoted atomic charges in the active Bax, 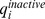 denoted atomic charges in the inactive Bax, and *n_res_* was the number of atoms in the residue. *ΔQ_res_* assessed the amount of charge that had been transferred to or from each residue. The second indicator was the root mean square deviation in atomic charge per residue:
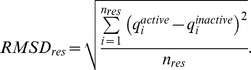

*RMSD_res_* assessed the intra-residue charge density redistributions.

### Sequence and Structural Alignment between Bax and Bak

The Bax/Bak sequence alignment was done for the UniProtKB/Swiss-Prot entries Q07812 (BAX_HUMAN) and Q16611 (BAK_HUMAN), and was performed using ClustalW2 with default parameters on the EBI server [63]. The structural models were visualized using VMD [64].

## Supporting Information

Figure S1 **Activator binding induces significant reorganization of intra-residue charge density in the functional regions of Bax.** Upon activator (Bim-SAHB) binding to Bax, significant reorganization of the intra-residue charge density is observed in the functional regions of Bax, suggesting that the activation is conveyed across the entire Bax molecule. The color coding from Figure 4 is maintained, with the C-domain in yellow, BH3 domain in cyan, central helix in green, the rest of active Bax in orange, and Bim-SAHB in purple. Additionally, the amino acid residues which suffer significant redistributions of their charge density are displayed explicitly (*RMSD_res_* one standard deviation higher than average; see Table S1). These residues can be found at the Bax activation site, on loop 1–2, inside the BH groove holding the Bax C-domain, and at the two ends of the C-domain itself. (A) Side view is given. (B) Top view of helix 5 is given.
(PDF)

Figure S2 **Sequence alignment between Bax and Bak reveals rather low sequence identity (ClustalW2 Score 19%).** An asterisk indicates a single, fully conserved residue. A colon indicates conservation between groups of strongly similar biochemical properties. A period indicates conservation between groups of weakly similar biochemical properties. The sequence alignment for the central helices and the structural alignment are given in Figure 6.
(PDF)

Table S1 **Changes in the charge profile of all Bax residues upon Bax activation.** Net charge transfer was computed as the total difference in charge per residue (*Q_res_*). The intra-residue charge density redistributions were evaluated as the root mean square deviation in charge per residue (*RMSD_res_*). Both descriptors were computed using EEM atomic charges, and their mathematical derivation can be found in the Methods section. All quantities are given in elementary charges (1 *e* has approximately 1.602×10^−19^ coulombs). The cell background colors mark the various domains of the Bax molecule in agreement with Figure 1 (BH3-domain in cyan, C-domain in yellow, loop 1–2 in pink, and helix 5 in green). The residues which exhibited a net charge transfer of more than one standard deviation over the average are marked in bold, and the color of the font indicates whether the respective residues became more positive (red) or more negative (blue) upon activation. These residues are also displayed explicitly in Figure 5.
(PDF)
