# Supplementary figures and images for: Charge Profile Analysis Reveals That Activation of Pro-apoptotic Regulators Bax and Bak Relies on Charge Transfer Mediated Allosteric Regulation
Source: PLoS Comput Biol. 2012 Jun 14;8(6):e1002565. doi: 10.1371/journal.pcbi.1002565 (PMC3375244; doi:10.1371/journal.pcbi.1002565)

**A**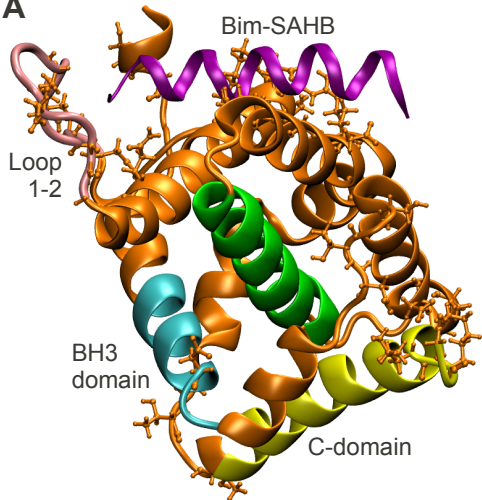**B**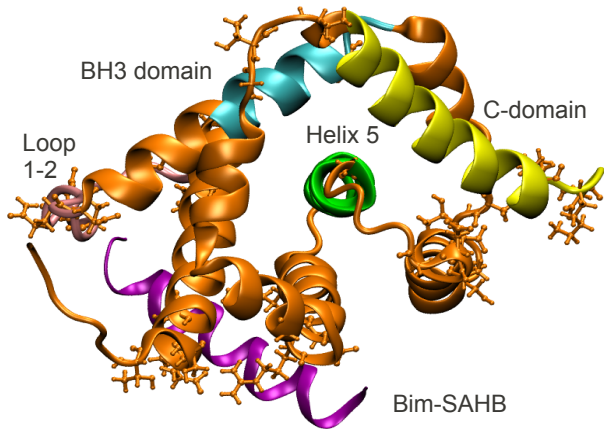

Supplement: Figure S1 — Activator binding induces significant reorganization of intra-residue charge density in the functional regions of Bax. Upon activator (Bim-SAHB) binding to Bax, significant reorganization of the intra-residue charge density is observed in the functional regions of Bax, suggesting that the activation is conveyed across the entire Bax molecule. The color coding from Figure 4 is maintained, with the C-domain in yellow, BH3 domain in cyan, central helix in green, the rest of active Bax in orange, and Bim-SAHB in purple. Additionally, the amino acid residues which suffer significant redistributions of their charge density are displayed explicitly (RMSDres one standard deviation higher than average; see Table S1). These residues can be found at the Bax activation site, on loop 1–2, inside the BH groove holding the Bax C-domain, and at the two ends of the C-domain itself. (A) Side view is given. (B) Top view of helix 5 is given. (PDF) [file pcbi.1002565.s001.pdf]
