# Supplementary material for: Charge Profile Analysis Reveals That Activation of Pro-apoptotic Regulators Bax and Bak Relies on Charge Transfer Mediated Allosteric Regulation
Source: PLoS Comput Biol. 2012 Jun 14;8(6):e1002565. doi: 10.1371/journal.pcbi.1002565 (PMC3375244; doi:10.1371/journal.pcbi.1002565)
Supplement: Figure S2 — Sequence alignment between Bax and Bak reveals rather low sequence identity (ClustalW2 Score 19%). An asterisk indicates a single, fully conserved residue. A colon indicates conservation between groups of strongly similar biochemical properties. A period indicates conservation between groups of weakly similar biochemical properties. The sequence alignment for the central helices and the structural alignment are given in Figure 6. (PDF) [file pcbi.1002565.s002.pdf]

```
Bax  1     - M D G S G E Q P - - - R G G G P T S - - - - - - - - - - - S E Q I M K   21
Bak  1     M A S G Q G P G P P R Q E C G E P A L P S A S E E Q V A Q D T E E V F R   36
               . * . *     *       .   *   * :                         : * : : : :

Bax  22    T G A L L L Q G F I Q D R A G R M G G E A P E L A L D P V P Q D A S T K   57
Bak  37    S Y V F Y R H Q Q E Q E A E G V A A P A D P E M V T L P L Q P S S T M G   72
           :   . :     :       * :     *     .       * * : .     * :     . : :

Bax  58    K L S E C L K R I G D E L D S - - N M E L Q R M I A A V D - - T D S P R   89
Bak  73    Q V G R Q L A I I G D D I N R R Y D S E F Q T M L Q H L Q P T A E N A Y  108
           : : . .   *     * * * : : :       :   * : *   * :     : :     : : . .

Bax  90    E V F F R V A A D M F S D G N F N W G R V V A L F Y F A S K L V L K A L  125
Bak  109   E Y F T K I A T S L F E S G - I N W G R V V A L L G F G Y R L A L H V Y  143
           *   *   : : * : . : * . . *   : * * * * * * * * :   * .   : * . * : .

Bax  126   C T K V P E L I R T I M G W T L D F L R E R L L G - W I Q D Q G G W D G  160
Bak  144   Q H G L T G F L G Q V T R F V V D F M L H H C I A R W I A Q R G G W V A  179
                 : .   : :     :     : . : * * :   . :   : .   * *   : : * * *   .

Bax  161   L L S Y F G T P T W Q T V T I F V A G V L T A S L T I W K K M G -        192
Bak  180   A L N - L G N G P I L N V L V V L G V V L L G Q F V V R R F F K S        211
```
