# Supplementary material for: Charge Profile Analysis Reveals That Activation of Pro-apoptotic Regulators Bax and Bak Relies on Charge Transfer Mediated Allosteric Regulation
Source: PLoS Comput Biol. 2012 Jun 14;8(6):e1002565. doi: 10.1371/journal.pcbi.1002565 (PMC3375244; doi:10.1371/journal.pcbi.1002565)
Supplement: Table S1 — Changes in the charge profile of all Bax residues upon Bax activation. Net charge transfer was computed as the total difference in charge per residue (Qres). The intra-residue charge density redistributions were evaluated as the root mean square deviation in charge per residue (RMSDres). Both descriptors were computed using EEM atomic charges, and their mathematical derivation can be found in the Methods section. All quantities are given in elementary charges (1 e has approximately 1.602×10^−19^ coulombs). The cell background colors mark the various domains of the Bax molecule in agreement with Figure 1 (BH3-domain in cyan, C-domain in yellow, loop 1–2 in pink, and helix 5 in green). The residues which exhibited a net charge transfer of more than one standard deviation over the average are marked in bold, and the color of the font indicates whether the respective residues became more positive (red) or more negative (blue) upon activation. These residues are also displayed explicitly in Figure 5. (PDF) [file pcbi.1002565.s003.pdf]

Table S1: Changes in the charge profile of all Bax residues upon Bax activation.

Residue	ΔQ_{res} [e]	$RMSD_{res}$ [e]	Residue	ΔQ_{res} [e]	$RMSD_{res}$ [e]	Residue	ΔQ_{res} [e]	$RMSD_{res}$ [e]
1	-0.1727	0.0197	**65**	**0.2925**	**0.0268**	129	-0.0789	0.0265
**2**	**-0.2581**	**0.0346**	66	0.0650	0.0267	130	-0.0329	0.0092
3	0.0362	0.0299	67	-0.0221	0.0207	131	-0.1535	0.0213
4	-0.1329	0.0471	68	-0.0845	0.0241	132	-0.0990	0.0195
5	0.1505	0.0473	69	0.1032	0.0322	133	-0.1109	0.0256
6	-0.0575	0.0233	70	0.0157	0.0276	**134**	**0.3244**	**0.0381**
**7**	**0.3395**	**0.0292**	71	-0.1625	0.0392	135	-0.0550	0.0148
8	0.0183	0.0252	**72**	**-0.2149**	**0.0342**	136	-0.0387	0.0149
9	-0.0806	0.0202	73	0.1549	0.0356	137	0.0572	0.0269
10	0.0749	0.0326	74	0.1090	0.0274	**138**	**-0.2163**	**0.0406**
11	-0.0580	0.0246	75	-0.0763	0.0341	139	-0.0770	0.0131
12	-0.0543	0.0261	76	-0.0212	0.0165	140	0.0605	0.0267
13	0.0429	0.0297	77	0.0882	0.0363	141	-0.1517	0.0251
**14**	**-0.2795**	**0.0387**	78	-0.1416	0.0198	**142**	**-0.3367**	**0.0535**
15	-0.0438	0.0341	79	0.0604	0.0217	143	0.0186	0.0133
16	0.1542	0.0440	80	0.1033	0.0273	144	-0.0900	0.0173
**17**	**-0.3526**	**0.0406**	81	-0.1483	0.0246	145	0.0221	0.0398
18	-0.1413	0.0249	82	-0.9790	0.0243	**146**	**-0.2237**	**0.0270**
19	0.1719	0.0214	83	0.0138	0.0282	147	0.1143	0.0314
20	0.0130	0.0180	**84**	**-0.3090**	**0.0427**	148	-0.0160	0.0172
21	0.0406	0.0406	85	-0.0824	0.0498	149	0.0015	0.0271
22	-0.0559	0.0172	86	-0.0585	0.0362	150	-0.0401	0.0419
23	-0.1198	0.0241	87	0.0218	0.0532	151	-0.1758	0.0307
24	0.0097	0.0160	88	-0.0281	0.0126	152	-0.0757	0.0271
25	-0.1363	0.0213	89	0.2063	0.0310	153	-0.0706	0.0350
26	0.0005	0.0112	90	-0.1491	0.0297	154	0.0215	0.0230
27	-0.0028	0.0198	91	0.0201	0.0149	155	0.1332	0.0161
28	0.0162	0.0234	92	-0.0622	0.0148	156	-0.0122	0.0235
29	-0.1682	0.0291	93	0.0009	0.0166	157	-0.0645	0.0239
30	0.0664	0.0141	**94**	**0.2583**	**0.0340**	158	-0.0152	0.0128
31	0.0481	0.0089	95	0.0280	0.0296	159	0.1476	0.0303
32	-0.0194	0.0407	96	0.0181	0.0154	160	-0.0065	0.0223
33	-0.1330	0.0359	97	-0.0047	0.0171	161	0.0606	0.0130
34	-0.1380	0.0351	**98**	**-0.2369**	**0.0458**	**162**	**0.2290**	**0.0255**
35	-0.0988	0.0221	99	-0.1696	0.0262	163	0.0564	0.0194
36	-0.0804	0.0285	100	-0.0130	0.0218	164	-0.0555	0.0214
**37**	**0.3251**	**0.0310**	**101**	**-0.2590**	**0.0408**	**165**	**0.3539**	**0.0289**
38	0.1516	0.0258	**102**	**-0.2960**	**0.0424**	166	0.1540	0.0518
39	0.1640	0.0394	103	0.0283	0.0381	**167**	**-0.3723**	**0.0508**
40	0.1234	0.0409	104	0.0962	0.0303	168	0.0442	0.0216
41	0.0227	0.0257	105	0.0431	0.0160	169	0.0308	0.0434
**42**	**-0.3192**	**0.05**	106	0.0669	0.0314	170	-0.0262	0.0273
43	0.0534	0.0228	**107**	**-0.2199**	**0.0328**	171	-0.0509	0.0123
**44**	**0.3388**	**0.0322**	108	0.1159	0.0517	172	-0.1682	0.0314
45	-0.0372	0.0322	**109**	**0.2676**	**0.0354**	173	0.1683	0.0231
**46**	**-0.2372**	**0.0589**	110	0.0538	0.0275	174	0.1826	0.0359
**47**	**0.3098**	**0.0319**	111	-0.0034	0.0239	175	-0.1683	0.0134
**48**	**-0.3366**	**0.0580**	112	-0.1253	0.0235	176	-0.0898	0.0191
**49**	**0.2935**	**0.0327**	113	-0.0981	0.0225	177	0.0877	0.0293
50	0.0295	0.04	114	0.1738	0.0182	178	0.0036	0.0167
51	-0.0329	0.0132	115	0.0333	0.0150	179	-0.0522	0.0175
52	0.0810	0.04	116	-0.1464	0.0131	180	-0.0921	0.0141
53	-0.0396	0.0454	117	0.0532	0.0165	181	-0.0433	0.0310
54	0.0887	0.0204	118	-0.0467	0.0269	182	-0.0004	0.0182
55	0.0890	0.0271	**119**	**0.2563**	**0.0326**	183	-0.0600	0.0184
56	0.0836	0.0255	120	0.0884	0.0186	184	-0.0403	0.0182
57	0.0399	0.0248	121	-0.0412	0.0119	185	0.1295	0.0210
58	-0.0536	0.0164	122	-0.0197	0.0154	186	0.1059	0.0190
59	0.0526	0.0141	123	0.0267	0.0157	187	-0.0701	0.0242
60	-0.0712	0.0321	124	-0.1912	0.0256	**188**	**-0.3319**	**0.0340**
61	0.0300	0.0186	125	-0.1811	0.0247	**189**	**0.3947**	**0.0493**
62	-0.0291	0.0187	126	0.1548	0.0251	**190**	**0.5118**	**0.0487**
63	0.0454	0.0159	127	0.1162	0.0216	191	-0.1540	0.0341
**64**	**0.3360**	**0.0315**	128	-0.0400	0.0154	192	0.0606	0.06
